# Introducing openESM: A database of openly available experience sampling datasets

**DOI:** 10.3758/s13428-026-03112-y

**Published:** 2026-07-16

**Authors:** Björn S. Siepe, Jonas M. B. Haslbeck, Matthias Kloft, Anabel Büchner, Yong Zhang, Eiko I. Fried, Daniel W. Heck

**Affiliations:** 1 Psychological Methods Lab, Department of Psychology, Philipps-Universität Marburg, Marburg, Germany; 2 Department of Psychological Methods, University of Amsterdam, Amsterdam, Netherlands; 3 Department of Clinical Psychological Science, Maastricht University, Maastricht, Netherlands; 4 Institute of Psychology, Humboldt-Universität zu Berlin, Berlin, Germany; 5 Department of Psychometrics and Statistics, Faculty of Behavioural and Social Sciences, University of Groningen, Groningen, Netherlands; 6 Department of Clinical Psychology, Leiden University, Leiden, Netherlands; 7 Department of Methodology & Statistics, Leiden University, Leiden, Netherlands

**Keywords:** Experience sampling, Daily life, Database, Time series, Generalizability

## Abstract

Experience sampling via mobile devices enables unprecedented insights into daily life. However, individual studies often cannot answer research questions conclusively, and open data are scattered across repositories in different formats. This impedes research into robustness, generalizability, and heterogeneity. We address this issue by introducing openESM, an open-source database of openly available experience sampling datasets in a harmonized format. The growing database currently comprises 60 datasets with more than 16,000 participants and more than 740,000 observations. Metadata can be searched via our website (openesmdata.org) to select and download datasets via packages in R and Python. We demonstrate the potential of openESM through an analysis of within-person correlations of positive and negative affect in 39 datasets, providing evidence for a large negative momentary correlation (-0.49, 95% CI: [-0.54, -0.42]). We end by discussing the design principles that will allow openESM to become a continuously evolving community resource for cumulative experience sampling research.

## Introduction

Research in various disciplines investigates how human behavior, experiences, and cognition unfold over time, how these processes differ between individuals, and how these differences relate to long-term outcomes such as mental health (Bolger et al., 2003; Browning et al., 2024; Shiffman et al., 2008). The advent of experience sampling approaches—the repeated data collection of subjective and, increasingly, physiological states in everyday life—has allowed us to answer these questions on a level of granularity that was previously unfeasible (Fritz et al., 2024). Such data are typically collected via smartphones, smartwatches, or other wearable devices.

As in social and behavioral sciences more broadly, research questions in experience sampling typically cannot be conclusively answered with one dataset from a single study (Yarkoni & Westfall, 2017). Instead, there is a need for replications and the joint analysis of multiple datasets to understand if effects are robust, and to what degree they depend on varying contexts (Baribault et al., 2018). This is particularly relevant in experience sampling research, given the resource-intensive nature of data collection, which typically limits the scope of individual studies. Self-reported, high-quality data requires a substantial and prolonged amount of time and effort from participants and researchers (Eisele et al., 2022). Meanwhile, the collection of passive data from wearable devices presents its own challenges, including device management, data handling, and privacy concerns (Onnela, 2021). As a result, the data used in most studies are often limited in terms of sample size, number of timepoints, participant diversity, and the measures employed (e.g., Wrzus and Neubauer, 2023). Thankfully, a growing number of experience sampling researchers have adopted open science practices by sharing their data and study materials. However, these are currently scattered across different repositories in various formats, making them difficult to find and reuse. This scattered landscape, combined with a focus on smaller individual studies, hinders research into daily life in general and work on robustness, heterogeneity, and generalizability in particular.

To address these challenges, we introduce openESM, a continuously updated database of openly available experience sampling datasets in a harmonized format. At the time of writing, it comprises 60 datasets, including 16,354 individuals and over 740,000 observations, covering a wide range of participants, designs, and constructs. The database includes longitudinal experience-sampling data from both observational and experimental studies, as well as passive-sensing data from smartphones and wearables, often combined with cross-sectional questionnaires. The longitudinal data were harmonized, which involves consistent variable naming, missing value coding, and file formatting across datasets (see Section “Methods” for details). openESM contains extensive, structured metadata for all datasets and variables. Both the data and metadata can be accessed, filtered, and searched on openesmdata.org such that researchers can easily find and select datasets relevant to their research questions. In addition, we provide direct interfaces via R and Python packages for convenient access to and handling of datasets.

In other scientific disciplines, database projects such as the UK Biobank (Sudlow et al., 2015), the Human Connectome Project in neuroscience (Marcus et al., 2011), or standardized benchmarks for methods in statistics and artificial intelligence (Bischl et al., 2025; Deng et al., 2009; Markelle et al., 2023; Olson et al., 2017) have allowed for remarkable innovations and scientific insights (Domingue et al., 2025). In psychology, similar efforts in recent years include a large openly available collection of datasets for applications of item response theory (Domingue et al., 2025), results from psychotherapy trials collected by the MetaPsy initiative (https://metapsy.com), data from confidence ratings across different research fields (Rahnev et al., 2020), and intensive longitudinal emotion datasets (Kalokerinos et al., 2026, see the Discussion section for a more detailed comparison to openESM). These resources enable highly powered studies and cumulative scientific progress. We hope that our database will bring similar advantages to experience sampling research.

The three main use cases for our database concern *substantive*, *design*, and *statistical* research questions. These are not necessarily mutually exclusive and inform each other. Within all of these, the broad scope of openESM will enable novel insights into heterogeneity and generalizability across large samples from diverse populations, sociodemographic attributes, and study designs.

At its core, most experience sampling research aims to answer *substantive* questions about the structure of psychological constructs or behavioral patterns in daily life and their associations and development over time. Recent cross-study analyses demonstrate the value of combining data from different studies for this purpose. For example, (Dejonckheere et al., 2019a) showed that affect dynamics had a limited utility in predicting psychological well-being beyond simpler summaries of affect, such as the mean or standard deviation. Relatedly, meta-analytical approaches across studies have been used to investigate whether the variability of affect is related to neuroticism (Kalokerinos et al., 2020; Mader et al., 2023).

Similarly, (Haslbeck et al., 2023) used data from multiple studies to establish robust empirical phenomena (multimodality and skewness of affective time series), and to relate heterogeneity in phenomena to participant and study characteristics. However, these studies require considerable effort to find, access, and harmonize all relevant datasets. Frameworks such as integrative data analysis (Curran & Hussong, 2009) and coordinated data analysis (Hofer & Piccinin, 2009) have outlined how cross-study synthesis enables cumulative knowledge generation, but rely on researchers having access to harmonized datasets. Beyond studying associations, the temporal nature of experience sampling further opens a natural window for a predictive perspective (Yarkoni & Westfall, 2017). This is relevant for studying how well theories and models can predict patterns in empirical data and for deriving potential just-in-time interventions (Nahum-Shani et al., 2018). In this context, investigating the generalizability of predictions is crucial for the practical relevance of predictive models, as illustrated by a multi-dataset study by Adler et al. (2022). openESM enables a wide variety of such studies with considerably less effort, thus allowing researchers to test the robustness and generalizability of findings and theories in a way that individual studies cannot achieve.

The wealth of new possibilities for data collection has given rise to many methodological questions about *design* of experience sampling studies, which has led to a surge of research in this area. This subsumes various research questions about how experiences are measured and how studies’ design choices influence their results. For instance, researchers have investigated the influence of different response scale formats on participants’ answers (Haslbeck et al., 2023), differences between studies with varying assessment frequencies or study durations (Haslbeck & Ryan, 2022; van Berkel et al., 2019; Wrzus & Neubauer, 2023), and the overlap of items assessed at different time scales (Leertouwer et al., 2022).

The integration of multimodal data, for example, from self-reports and passive sensors, also requires measurement research on their alignment and differences (Siepe et al., 2025; Velozo et al., 2024). Such research especially profits from jointly analyzing multiple datasets that vary in study design features such as response scale levels, measurement frequency, or questionnaire length, possibly explaining heterogeneity in findings across studies and informing future design choices.

Experience sampling data poses many analytical challenges, which have prompted the development of many novel *statistical* methods for intensive longitudinal data. A large database such as *openESM* can benefit this research in multiple ways. First, it enables the investigation of common patterns such as multimodal distributions or floor effects in time series (Haslbeck et al., 2023; Muthén et al., 2025) across studies, which can in turn inspire the development of novel statistical models. Second, the empirical properties of the data can inform the design of simulation studies investigating statistical properties of different methods with artificially generated data (Siepe et al., 2024). This is also relevant for data-generating settings in simulation-based power analysis, a topic crucial for planning new experience sampling studies (Lafit et al., 2021; Revol et al., 2024; Zhang et al., 2025). Third, openESM datasets can be used for benchmarking experiments, in which the predictive accuracy of different methods is compared using real data (Weber et al., 2019). This practice has been widely adopted across statistical disciplines and has led to the creation of many standardized benchmarks in other fields (see, e.g., Olson et al., 2017; Deng et al., 2009; Markelle et al., 2023). Such a predictive approach using empirical data is important to reveal practical differences between methods in real applications. Fourth, researchers can study conditions under which different methods reach similar or different conclusions, thereby improving the understanding of the sensitivity of empirical findings to different analysis choices (Steegen et al., 2016). The breadth of openESM can thus enhance the quality of methodological research by grounding it in real-world characteristics, thereby inspiring new developments and generating more useful knowledge for applied researchers.

In what follows, we first give an overview of the openESM database and its functionality. We then illustrate how easy it is to perform a large reanalysis using our database, with the example of the within-person association between negative and positive affect. This analysis fits particularly well to demonstrate our database, as emotions are fundamental to many experience sampling studies across specific fields of research. This question also exemplifies the intersection of all three main use cases of our database. From a *substantive* perspective, the extent of this momentary association has been debated for a long time and is of high theoretical relevance for theories of affect (Dejonckheere et al., 2018; Larsen et al., 2017). However, *design* characteristics of studies, such as the sampling frequency or the specific affect items, might influence heterogeneity in these within-person couplings across datasets. Finally, estimating within-person associations and aggregating them across multiple datasets involves several *statistical* decisions that can influence the results. We do not aim to provide a conclusive answer to these questions, but rather use the example to demonstrate the potential of openESM for addressing these three main use cases.

We conclude by discussing novel research opportunities facilitated by openESM and outline our plans for extending its functionality in the future. The database’s technical details and design principles, along with the details of our example analysis, are described in the “Methods” section.

## Results

**Fig. 1:**
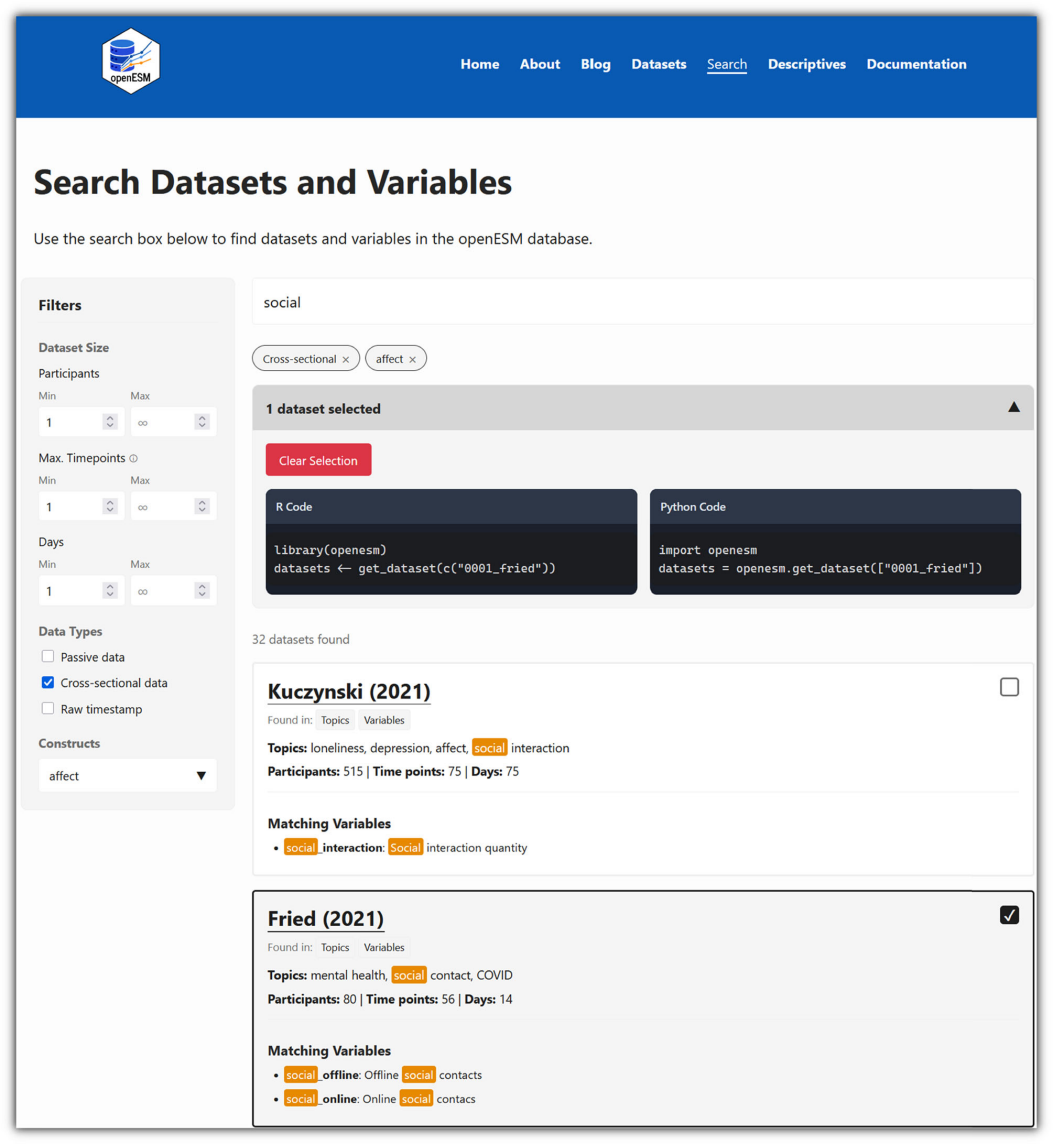
Screenshot of the openESM search functionality showcasing filtering, keyword search, and automatic code generation, which enables users to access datasets via these programming languages directly

Users can access openESM via our web interface, which provides usage instructions, a dataset overview, detailed documentation, and a search interface. As shown in Fig. 1, users can filter datasets by dataset characteristics such as minimum sample size or number of observations, select constructs of interest, and search for keywords (here: “social”). The interface displays matching datasets with key metadata and automatically generates R and Python code for downloading the selected datasets.

**Fig. 2:**
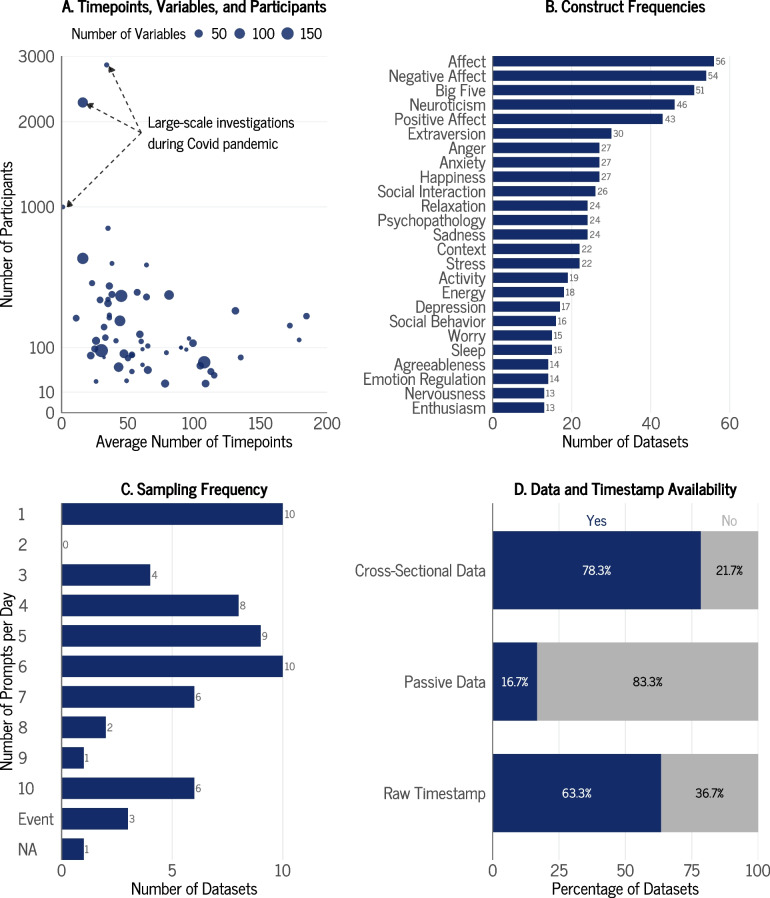
Descriptive statistics summarizing openESM. **A** the average number of non-missing observations (*x*-axis), the number of participants (*y*-axis, square-root scale), and the number of variables (size of points) for each dataset. **B** the number of datasets (*x*-axis) that contain a given construct in the ESM data (*y*-axis). **C** the number of datasets (*x*-axis) with a certain sampling frequency (*y*-axis), where "Event" represents event-contingent sampling. **D** Summary of the percentage of datasets (*x*-axis) that contain cross-sectional data, passive data, and raw timestamps (*y*-axis)

### Overview of openESM datasets

**Fig. 3:**
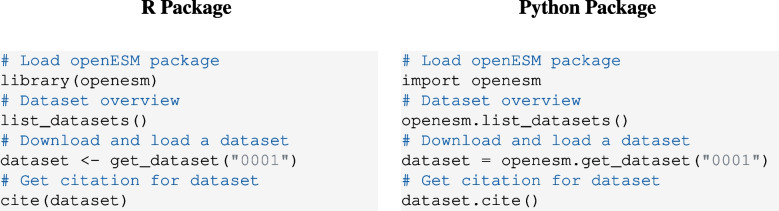
openESM package usage examples. get_dataset accepts both the dataset number (e.g., "0001") and the combination of dataset number and author name as shown in Fig. 1 (e.g., "0001_fried")

Currently, the database contains 60 datasets with 16,259 participants (median 122, range 20–2855). The median number of variables in a dataset is 25 (range: 8 - 178). In panel A of Fig. 2, we show the number of timepoints, number of participants, and variables for each dataset. The average number of timepoints per participant is spread widely. Some datasets have a very low average observation number, as they include many individuals with only one observation. Most studies have fewer than 500 participants, with some noticeably large outliers. In panel B, we show the most common constructs to which these variables can be assigned. The large majority of datasets contain variables measuring some form of affect or personality. In panel C, we display the sampling frequency of datasets. It ranges from once per day to ten times per day. Panel D contains information about the availability of cross-sectional data (e.g., demographics or personality traits), passive data, and raw timestamps. The large majority of studies (*n* = 47, 78.3%) included in our database also provide cross-sectional data, whereas only a relatively small minority contain passive data (*n* = 10, 16.7%). Raw timestamps, relevant for fine-grained modeling of changes over time, are available in around two-thirds of the datasets (*n* = 38, 63.3%). As evident in all panels, the datasets display considerable heterogeneity. The references to all primary publications are listed and highlighted in the References section of our article. We show example code snippets to download a selected dataset via our R and Python packages in Fig. 3.

### Example analysis: Estimating the within-person association of positive and negative affect

In our example, we demonstrate how openESM facilitates large-scale analyses across experience sampling datasets. We were interested in the within-person association of positive and negative affect for each individual and in understanding how this association varies across datasets. To model this association, we aggregated positive and negative affect into person-specific sum scores at each timepoint, estimated the within-person correlation across timepoints, and then performed a hierarchical meta-analysis on these correlations to account for the nesting of correlations in datasets. All online supplements, including the code and data to reproduce our results, are available at github.com/openesm-project.

**Fig. 4:**
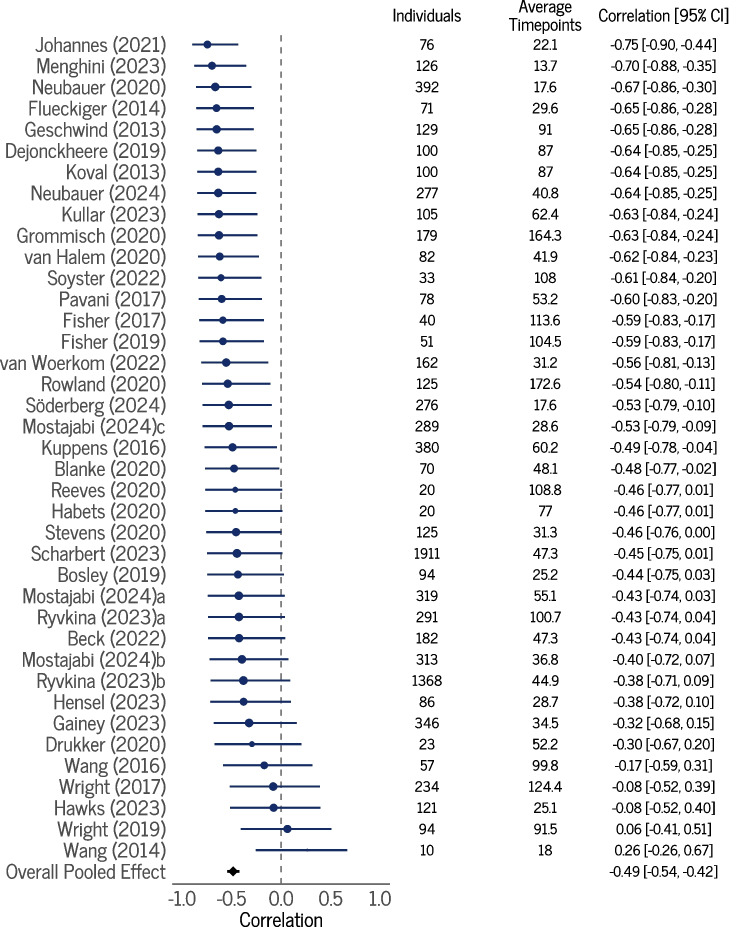
Within-person correlation of positive and negative affect across datasets. *Points* indicate the point estimate of average within-person correlations within a study, with the point size representing the weight of the estimate for the overall effect, and *lines* indicating 95% confidence intervals. The latter are derived from the multilevel meta-analytic model and account for within-dataset and between-dataset uncertainty

The first step of this analysis was using the search functionality on the website to find datasets containing items related to positive and negative affect. Across the 39 datasets with 8456 individuals and 529,203 timepoints that contained measures of positive and negative affect, positive and negative affect correlated negatively with a pooled correlation of -0.49 (95% CI: [-0.54, -0.42]). Across datasets, this correlation varied from -0.75 to 0.26. This means that, at moments when individuals reported higher positive affect, such as feeling happy or enthusiastic, they tended to simultaneously report lower negative affect, such as feeling sad or anxious, and vice versa. Figure 4 illustrates this association for all datasets and a meta-analytic pooled association in a forest plot. This analysis shows a moderate-to-strong negative correlation between positive and negative affect, suggesting that they are often inversely related, with considerable heterogeneity across datasets that warrants further investigation.

Regarding study design, we used the number of affect items, the number of prompts per day, and the response scale type (Likert vs. visual analog scale) as predictors of the strength of the within-person association. We found a significant effect of the number of prompts per day on the strength of within-person correlations, with each additional prompt being associated with a -0.04 (95% CI: [-0.07, -0.01]) change in correlation. Other effects were non-significant (see the supplement for more details). This finding illustrates how design choices regarding the temporal granularity of assessments may be linked to differences in empirical findings, with more experimental research needed to substantiate a causal link (see, e.g., Hasselhorn et al., 2022).

Next, we investigated the robustness of our findings in light of different statistical analysis choices. We applied 12 alternative preprocessing methods (different detrending and handling of missing data) and modeling approaches (Spearman or Pearson correlation). Our overall findings were robust to these alternatives, with aggregated effects ranging from -0.48 to -0.53 (median = -0.50, SD = 0.02). On average, Pearson correlations were more strongly negative with a difference of -0.05 compared to Spearman correlations. This finding implies that while the choice of correlation measure does matter somewhat, our overall findings are robust across a range of reasonable analysis choices. Additional details on all parts of our example analysis are available in our online supplement.

## Discussion

The openESM database addresses core challenges of experience sampling research: individual studies can only provide limited evidence about the generalizability of findings across different populations, research designs, and methodological choices. By combining and harmonizing data from more than 16,000 individuals from 60 studies, we enable large-scale, cumulative experience sampling research.

We showcased these novel possibilities in our example analysis on more than half a million timepoints, by far the largest version of this analysis so far. We found a robust negative concurrent within-person correlation between positive and negative affect, which was stronger for datasets with higher sampling frequencies and robust across different statistical choices. As emphasized before, we provide this simple analysis as a starting point for future in-depth analyses. Future *substantive* research could investigate a potentially nonlinear relationship between positive and negative affect (Vanhasbroeck et al., 2024), or examine their association in relation to depression or emotional differentiation to study its association with psychopathology (Dejonckheere et al., 2018) and stable between-person differences. Additional *design* features of the studies that we used, such as recruitment strategies or financial incentives for participation, could also be used to investigate heterogeneity in the within-person couplings. *Statistical* research could build on our example by investigating different ways of computing person-specific scores for affect, ranging from sum scores (which we used) to factor scores and latent variable models, which might influence the strength of their correlations. Relevant empirical properties observable across datasets, such as the distribution of within-person correlations (Fig. 4), typical sample sizes, and the number of timepoints, could also directly inform data-generating settings in simulation studies or power analyses for future experience sampling research.

The breadth of openESM enables research into a wide variety of topics across different substantive fields, such as the combination of passive sensing and self-report data (nine datasets), relating cross-sectional to experience sampling data (47 datasets), or investigating the influence of smartphone and/or social media use (eight datasets). The 60 studies vary considerably in their measurement approaches, item wordings, and sample characteristics. This enables fine-grained research into how design differences unfold in a study’s results. Similarly, the heterogeneity in dataset characteristics paired with the homogeneous formatting of the metadata and datasets enables statistical research to base simulated data on realistic settings and to conduct large-scale benchmarking studies comparing different statistical methods. Our database makes this type of research synthesis very easy: Instead of having to engage in a multi-month research project to request, find, clean, and harmonize existing datasets, such harmonized data can now be accessed within minutes using the web and R and Python interfaces.

A central goal of our project was to design a database that balances user-friendliness, sustainability, and community involvement. Datasets and metadata are stored in permanent, open repositories on Zenodo independent of our continued maintenance of the project, while our standardization of metadata and centralized search functionality ensure that they remain findable, reusable, and comparable across studies. Our openly available software packages lower the barrier for reuse, providing researchers with licensing information, references, and versioned (meta-)data for long-term reproducibility. Clear contribution guidelines for both datasets and software development, combined with our fully open-source approach, enable further developments based on user needs.

Our database complements other community tools in experience sampling research. To enhance the usefulness of metadata, openESM could be connected to the ESM Item Repository (Kirtley et al., 2018), a collection of over 3000 items used in experience sampling studies. This could improve the level of detail in the metadata of openESM, while providing researchers with immediate access to the data they are interested in. The EMOTE database (Kalokerinos et al., 2026), initially focused on emotion data, also contains valuable datasets for experience sampling studies in general. openESM adopts a fully open-source structure, whereas EMOTE requires obtaining permission for data access and reuse. These differences in scope and design can create a synergy and make EMOTE and our openESM database complementary: researchers can develop and test analyses and methods using openly available openESM data, and then validate findings using EMOTE’s permission-controlled datasets.

We are actively working on improving and extending openESM in several ways. First, the complex and heterogeneous nature of experience sampling datasets typically requires researchers to read all the available documentation and original papers for the datasets we provide. Researchers need to understand study-specific context because information that may currently be lacking from our metadata could explain diverging results across datasets. However, as openESM is an open-source resource, researchers interested in collecting specific information can easily contribute more detailed metadata to the database. We have designed openESM in a way that makes such extensions easy, and we are actively working on adding more metadata.

Second, the heterogeneity of experience sampling datasets described above impedes centralized and automated large-scale analyses across many variables and datasets, as is commonly done in meta-analysis (Harrer et al., 2022) or machine learning (see, e.g., Bischl et al., 2025). We nevertheless believe that standardized reanalyses and benchmarking projects could be worthwhile for experience sampling research. For example, as in other benchmarking projects (Qiu et al., 2024), comparisons of forecasting models could be automated such that developers of novel methods only need to provide their model’s function to a generic wrapper, and its performance is automatically evaluated and compared to other methods. The results of common (re-)analyses, such as forecasting benchmarks or meta-analyses, could be stored in an accessible format to enable easy extensions for further research while saving computational overhead.

Third, although we have collected a large and diverse set of data, there is clearly a bias in terms of which researchers share their data and which populations are included. For example, most data were collected in WEIRD (Western, Educated, Industrialized, Rich, Democratic, Henrich et al., 2010) countries, potentially threatening generalizability to other populations. Additionally, data sharing of clinical populations may be more difficult and raise more concerns about data privacy (Walsh et al., 2018). We are working actively to mitigate this issue through community engagement in sharing more diverse datasets. Still, we believe it will likely remain a limitation of analyses based on openly available data in the foreseeable future.

Experience sampling research needs larger datasets to facilitate analyses of robustness, generalizability, and heterogeneity across studies, designs, contexts, and populations. The openESM database has the potential to enable all of that, thus leading to more comprehensive and large-scale experience sampling research by providing an open-source resource with freely available datasets and rich metadata. We invite researchers to contribute their data and expertise to further expand the database and its software architecture, thereby making it a continuously evolving community resource. Instructions for doing so are available on our website (openesmdata.org).

## Methods

In the following, we describe the overarching design philosophy of openESM, our data handling, the backend and frontend of our database, and the main methodological decisions in our example analysis.

### Design philosophy

The guiding principle in creating the openESM database was that all (meta-)data should be as Findable, Interoperable, Accessible, and Reusable (the FAIR principles, Wilkinson et al., 2016) as possible. We oriented ourselves at databases and community standards in other fields, such as the brain imaging data structure (BIDS, Gorgolewski et al., 2016) in neuroimaging or the Penn machine learning benchmark suite (Olson et al., 2017). Based on our experience with experience sampling data, we preprocessed and annotated the data to maximize the ease of reuse for different researchers and research questions. We describe our main design decisions, which are based on these principles, below; for a comprehensive overview, please refer to our documentation at openesmdata.org.

### Datasets

#### Collection process

Some of the authors of this article have collected references to openly available datasets for some time (see Haslbeck et al., 2023, for a previous smaller collection). Beyond these, we employed an opportunistic sampling approach to identify further ESM datasets by contacting colleagues and searching the Open Science Framework and Zenodo for relevant data. This process was not designed to be exhaustive but represents the starting point for a continuously evolving resource. Each dataset either has a permissive license for reuse, or we asked the data owners for their permission to re-upload the data to our database with a Creative Commons Attribution Noncommercial license (CC-BY-NC 4.0). For each dataset, we provide reuse license information and at least one required reference that needs to be cited for reuse. Details about ethical approval for each dataset are available in the original publications listed within our metadata.

#### Inclusion criteria

Datasets were included based on four minimal criteria: (1) a minimum of 20 participants to enable group-based modeling approaches, (2) at least 20 possible measurement occasions per participant to support person-specific model estimation, (3) inclusion of at least two self-report variables measured longitudinally to align with the project’s focus and enable multivariate modeling strategies, and (4) exclusion of synthetic or simulated data to ensure authentic human data.

#### Data processing

We annotated metadata for each dataset and extracted variable information from codebooks, documentation, and previous publications. We provide an overview of the structure of dataset-level and variable-level metadata in Table 1. The metadata are stored in JavaScript Object Notation (JSON) to ensure machine readability. All datasets were converted to tab-separated value (.tsv) format to ensure compatibility across programming languages. Both of these choices resemble the BIDS neuroimaging standard (Gorgolewski et al., 2016). Each dataset includes standardized columns for participant ID, beep number, and day number to facilitate merging and analysis across datasets.

**Table 1:** Overview of dataset-level metadata fields in openESM

Category	Field	Description
General Info	Dataset ID	Unique dataset identifier in the openESM database
	Author	First author of the dataset
	Year	Year of publication
	Reference A	Primary publication reference
	Reference B	Secondary publication reference (if applicable)
Code & Data	Link to Zenodo	DOI link to harmonized dataset on Zenodo
	Paper DOI	DOI of associated paper
	Link to data	Direct URL to raw original dataset files
	Link to Code	Link to original analysis code
	Link to Codebook	URL to variable documentation
	License	Data usage license
Design & Participants	N Participants	Number of study participants
	N Timepoints	Max. number of possible ESM observations
	N Days	Number of days of data collection
	N Beeps/Day	Number of prompts per day
	Passive data	Availability of passive data
	Which passive data?	Types of passive data collected
	Cross-sectional	Availability of cross-sectional/trait measures
	Topics	Primary topics of study
	Implicit Missingness	Whether missing observations implicitly missing
	Raw Time Stamp	Availability of timestamps
	Sampling Scheme	ESM prompt schedule
	Participants	Participant characteristics
Variable-Specific Metadata	Name	Variable name in dataset
	Description	Brief description
	Variable Type	Data type (numeric, date, etc.)
	Details	Wording of questions or other details
	Labels	Scale labels and response options
	Transformation	Any applied data transformations
	Source	Origin of the variable (e.g., questionnaire)
	Assessment Type	Method of data collection (ESM/Daily/Passive)
	Construct	Construct measured
	Comments	Additional notes

We opted for construct annotation to make it easier to search our database. The constructs that we specified may not align with the original authors’ intentions

During data cleaning, we attempted to harmonize the datasets as much as possible by, among other steps, ensuring consistent variable names, removing redundant aggregate columns, verifying the consistent coding of missing values, and implementing consistent formats for time and date variables. We also ensured that all datasets contained harmonized columns indicating the participant ID, the day, and the number of the day’s prompt. All data cleaning scripts are available at github.com/openesm-project/openesm-cleaning. We also assigned each variable to one or multiple constructs to facilitate easier searching. While we also collected available cross-sectional and passive sensor data components (or maintained links to them), we have not yet systematically cleaned and harmonized these data. As they possess the same unique participant ID, they can already be merged and cleaned by researchers interested in them.

### Architecture

**Fig. 5:**
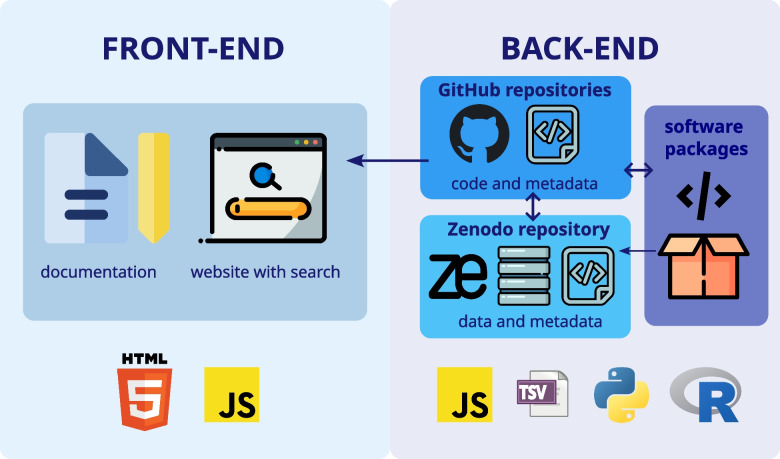
Components of the openESM database. Technology icons used in this figure come from the following sources: HTML5 logo (CC BY 3.0, W3C, 2011), JavaScript logo (MIT License, Williams, 2011), R logo (CC BY-SA 4.0, R Core Team, 2016), Python logo (GPL, Python Software Foundation, 2005), TSV file icon (CC BY-SA 4.0, Masao, 2007), Zenodo logo (CC BY-SA 4.0, CERN, 2013), and GitHub logo (MIT License, Github, Inc., 2013). All other icons were taken from https://www.svgrepo.com with a CC0 license

We provide an overview of the openESM architecture in Fig. 5. We outline the major design elements and choices below; further details are available in the online documentation.

#### Backend

All datasets are stored in a Zenodo community (zenodo.org/communities/openesm/). We chose Zenodo (European Organization For Nuclear Research, 2013), as similar repositories (e.g., Godahewa et al., 2021) have done, because of its safe storage, version control, citability via digital object identifiers (DOIs), possibility for data upload by other researchers, and its seamless API access, which enables incremental updating and downloading. We provide a detailed contribution guide on our website (openesmdata.org) for researchers wishing to contribute their data.

Additionally, we store all metadata in the project’s GitHub repository, which enables the community to propose changes or additions to the metadata via pull requests. New versions on GitHub are synchronized with Zenodo. This allows for consistent versioning of updates to the metadata and gives users a versioned reference to the particular instance of the database used in a project, aiding the long-term reproducibility of projects using openESM.

We created an R package (available at github.com/openesm-project/openesm-r) and a Python package (pypi.org/project/openesm) with largely identical helper functions to access the (meta-)data and to obtain license information and references for each dataset. When selecting datasets in the search function of our website, code to access the subset of datasets in both R and Python is automatically generated. Further documentation of the packages is available at openesmdata.org.

#### Frontend

Our website offers the most user-friendly access to the database. It provides an introduction to the database, explaining its use and the requirements for its users. Users can browse through all datasets and access all metadata. The website was developed in html and JavaScript. As shown in Fig. 1, we provide an advanced search tool that enables filtering datasets and searching for keywords and code to download the selected datasets.

### Details about example analysis

To decide whether a dataset was relevant for our example analysis, we needed to categorize items as inquiring about positive or negative affect. There is considerable heterogeneity in how affect is measured within the literature (Cloos et al., 2023). Items were therefore selected based on agreement among the co-authors. A list of the items, alongside our rationale for including or excluding some of them, is provided in the online supplement; 39 out of 60 datasets contained at least two items for positive and negative affect each. We provide a list of these datasets in our online supplement. They contained between four and 31 items for affect and 10,960 participants in total. We excluded participants with fewer than ten valid timepoints in our analysis, which is an arbitrary cutoff. We also excluded participants with zero variance in either of the affect variables, and participants for whom the sampling variance of the estimated correlation was infinite (due to boundary values). This left us with a final sample size of 8457 participants.

To avoid spurious correlations induced by temporal trends in the variables, we removed a linear trend from the variables by regressing them on a time variable and using the residuals of this regression as data for subsequent analyses. If no time variable was available (which was the case for two datasets), we did not perform detrending.

We first estimated the Pearson correlations between person-mean-centered sum scores of positive and negative affect for each individual. We used a mixed-effects meta-analysis approach to obtain an aggregated correlation estimate and account for nesting in studies. To do so, we applied a Fisher-*z* transformation to correlations as in meta-analysis. We then conducted the meta-analysis with individuals nested in datasets using the R package metafor (Viechtbauer, 2010). For ease of interpretation, we transformed all results back to the scale of correlations.

We then predicted the correlations with study design characteristics in a meta-regression. We used the combined number of affect items, the number of response options for the individual items, and the response scale type (Likert vs. visual analog scale) as predictors.

To investigate the robustness of conclusions to different statistical choices, we varied the detrending strategy (detrending/differencing), the choice of correlation measure (Pearson/Spearman), and the cutoff for excluding individuals with too few datapoints (5/10/20 observations). In each of the 12 possible combinations of these analysis steps, we re-estimated our main meta-analysis. This additional analysis not only allows us to understand how robust our overall results are to different analytical choices, but could also enable understanding in which contexts alternative specifications might be more impactful. It thus advances both knowledge of the content matter and of analysis choices.

### Open practices

All datasets and metadata are available via the openESM website (openesmdata.org) and permanently archived on Zenodo (zenodo.org/communities/openesm/). We provide all code for data cleaning, the database, and the analyses in this paper under github.com/openesm-project. The repository for the analyses in this paper also contains a Makefile and a Docker container (Merkel, 2014) to enable computational reproducibility (github.com/openesm-project/openesm-paper). The version of the openESM metadata used in this article is 1.0.0. Besides references cited elsewhere in the manuscript, these are the original references of articles associated with the datasets in openESM:

## Data Availability

All datasets and metadata are available via the openESM website (https://openesmdata.org) and permanently archived on Zenodo (https://zenodo.org/communities/openesm/).

## Declarations

### Code availability

We provide all code for data cleaning, the database, and the analyses in this paper on GitHub (https://github.com/openesm-project). The repository for the analyses in this paper also contains a Makefile and a Docker container (Merkel, 2014) to enable computational reproducibility (https://github.com/openesm-project/openesm-paper).
